# Immune‐mediated disruption of the blood–brain barrier after intracerebral hemorrhage: Insights and potential therapeutic targets

**DOI:** 10.1111/cns.14853

**Published:** 2024-07-21

**Authors:** Peijun Jia, Qinfeng Peng, Xiaochong Fan, Yumeng Zhang, Hanxiao Xu, Jiaxin Li, Houn Sonita, Simon Liu, Anh Le, Qiongqiong Hu, Ting Zhao, Shijie Zhang, Junmin Wang, Marietta Zille, Chao Jiang, Xuemei Chen, Jian Wang

**Affiliations:** ^1^ Department of Pain Medicine The First Affiliated Hospital of Zhengzhou University Zhengzhou China; ^2^ Department of Human Anatomy School of Basic Medical Sciences of Zhengzhou University Zhengzhou China; ^3^ School of Life Sciences Zhengzhou University Zhengzhou China; ^4^ David Geffen School of Medicine University of California Los Angeles Los Angeles California USA; ^5^ George Washington School of Medicine and Health Sciences Washington DC USA; ^6^ Department of Neurology Zhengzhou Central Hospital Affiliated to Zhengzhou University Zhengzhou Henan China; ^7^ Department of Neurology People's Hospital of Zhengzhou University Zhengzhou China; ^8^ Division of Pharmacology and Toxicology, Department of Pharmaceutical Sciences University of Vienna Vienna Austria

**Keywords:** blood–brain barrier, intracerebral hemorrhage, immune cell, cytokines, tight junction proteins

## Abstract

**Aims:**

Intracerebral hemorrhage (ICH) is a condition that arises due to the rupture of cerebral blood vessels, leading to the flow of blood into the brain tissue. One of the pathological alterations that occurs during an acute ICH is an impairment of the blood–brain barrier (BBB), which leads to severe perihematomal edema and an immune response.

**Discussion:**

A complex interplay between the cells of the BBB, for example, pericytes, astrocytes, and brain endothelial cells, with resident and infiltrating immune cells, such as microglia, monocytes, neutrophils, T lymphocytes, and others accounts for both damaging and protective mechanisms at the BBB following ICH. However, the precise immunological influence of BBB disruption has yet to be richly ascertained, especially at various stages of ICH.

**Conclusion:**

This review summarizes the changes in different cell types and molecular components of the BBB associated with immune‐inflammatory responses during ICH. Furthermore, it highlights promising immunoregulatory therapies to protect the integrity of the BBB after ICH. By offering a comprehensive understanding of the mechanisms behind BBB damage linked to cellular and molecular immunoinflammatory responses after ICH, this article aimed to accelerate the identification of potential therapeutic targets and expedite further translational research.

## Graphical abstract

Blood–brain barrier disruption and immune system infiltration after intracerebral hemorrhage.

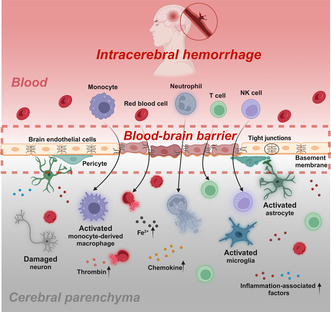

## 1 INTRODUCTION

Intracerebral hemorrhage (ICH) is a result of the rupture of blood vessels in the cerebral parenchyma. The second most common subtype of stroke follows a high incidence rate and mortality with limited treatment options.^1^, ^2^ Neuroinflammation and the disruption of the blood–brain barrier (BBB) are two hallmarks of secondary brain damage after ICH.^3^ BBB injuries triggered a cascade of changes involving immune responses, the degradation of tight junction proteins (TJPs), and increased trans‐endothelial permeability. The extravasation of blood components, including thrombin, fibrinogen, and clot‐derived degradation products of red blood cells, precipitates an extensive immune‐inflammatory response in the perihematomal area. This response induces pronounced cytotoxic and vasogenic edema, culminating in adverse outcomes. Currently, clinical research in the field of drug treatment for ICH primarily centers around the exploration and creation of novel drugs targeting the injury mechanisms of this condition. Several internal mechanisms have been identified that can potentially ameliorate the pathology associated with the permeability of the BBB. However, the outcomes of these therapeutic approaches have yet to yield the desired effectiveness, and research on the BBB after ICH is still limited.^4^

Activated immune cells around the hematoma stimulate the expression of pro‐inflammatory factors, leading to peripheral leukocyte infiltration and increasing levels of cytokines and chemokines.^5^ These immune cells also activate matrix metalloproteinases (MMPs), which subsequently compromise the integrity of BBB and facilitate the recruitment of peripheral immune cells to the affected regions. The subsequent damage to the BBB directly results from this sequence of events. It is pertinent to note that neuroinflammation, in its initial stages, significantly contributes to brain injury in cases of ICH. Nevertheless, the resultant inflammatory alterations in neutrophils, macrophages, and astrocytes may facilitate recovery during later stages. This involves resolving inflammation and promoting neurogenesis, angiogenesis, and neuronal plasticity. Moreover, immune cells in the central nervous system (CNS) communicate with peripheral immune cells, creating a complex inflammatory network that may indirectly impact BBB integrity. Therefore, a comprehensive understanding of the transition within the neuroinflammatory response mechanism from injury to repair and the correlation between the cellular and molecular immune‐inflammatory response to the disruption in BBB may aid in identifying potential therapeutic targets for ICH.

This article aims to provide an overview of the current knowledge regarding the mechanisms that underlie the disruption of the BBB associated with immune‐inflammatory responses, with a determination of the interactions between cerebral and peripheral immune cells after ICH. We provide a high‐level overview of the structural characteristics of the BBB and the various factors that can potentially affect its integrity. We explore the impact of different cellular and molecular immune components, such as cytokines, chemokines and adhesion molecules, and MMPs, as well as microglial activation and peripheral immune cells. Finally, we explore recent therapeutic advancements and emerging 3D tissue engineering approaches that aim to address BBB disruption and serve as a reference for future research and clinical transformation.

## 2 DISRUPTION OF BBB COMPONENTS POST‐ICH AND IMMUNOLOGICAL IMPACTS

**FIGURE 1:**
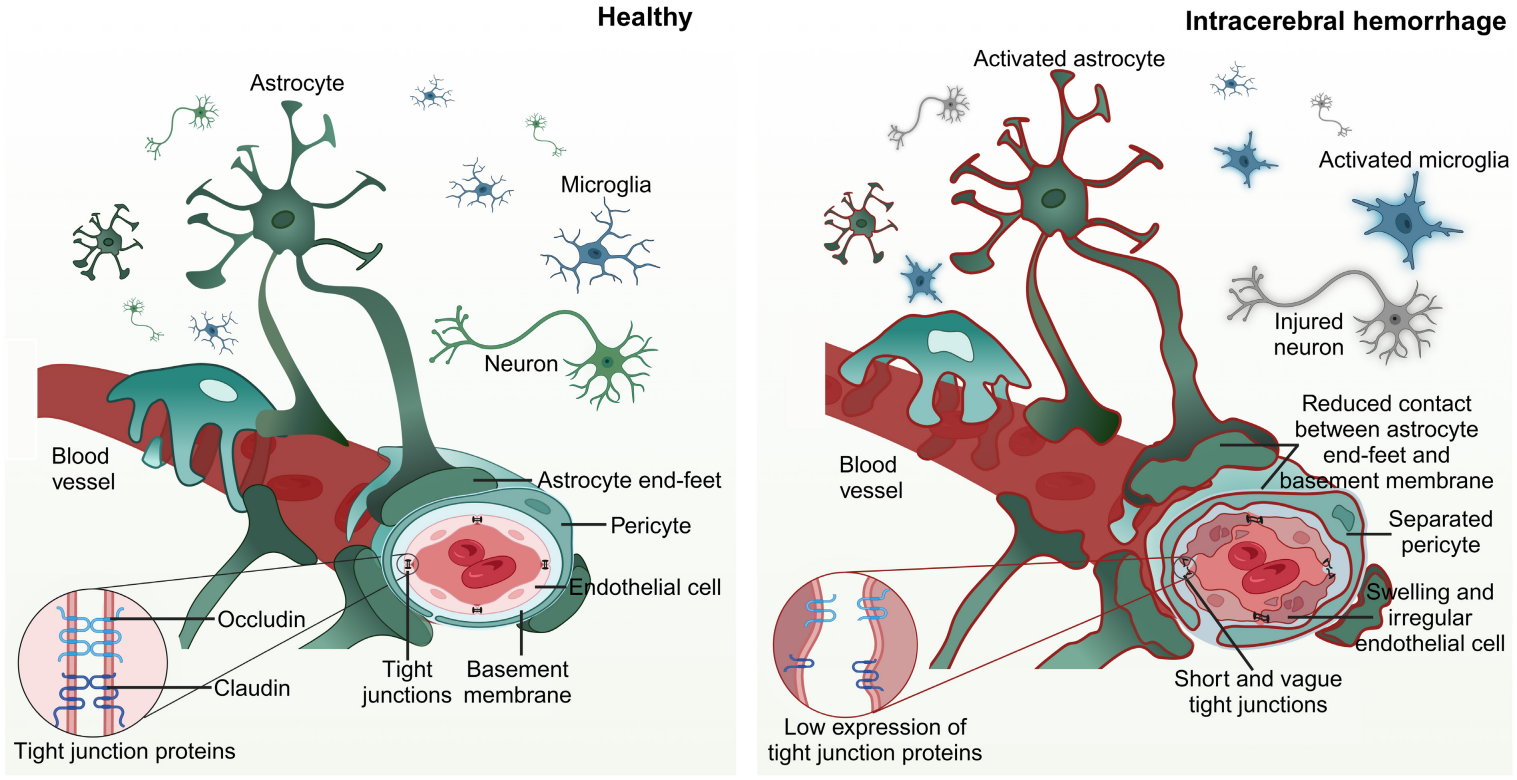
The structure of the neurovascular unit in healthy and post‐ICH conditions. The neurovascular unit comprises brain endothelial cells (BECs), basement membrane (BM), pericytes, astrocytes, microglia, and neurons. BECs are the main component of the blood–brain barrier (BBB) and are arranged regularly and tightly connected by tight junction proteins (TJPs). Surrounding the BECs are the BM, pericytes, and astrocyte end feet, which control the passage of cells and molecules into and out of the brain. When an intracerebral hemorrhage (ICH) occurs, the structure of the BBB is compromised, leading to swollen and irregular BECs, reduced expression of TJPs, and shortened and unclear tight junctions, resulting in increased BBB permeability. In addition, pericytes and astrocyte end feet show reduced contact with the BM, and toxic components in the hematoma following ICH activate astrocytes and microglia, damaging neurons.

Maintaining the integrity of the BBB is crucial for ensuring the correct structural and functional connections in the brain, synaptic linkages, and the exchange of information among various cell types within the neurovascular unit. The BBB is composed of complex interactions of different components, including brain endothelial cells (BECs), pericytes, astrocytes, cellular junctions, and the basement membrane (BM).^6^, ^7^ The CNS has a low level of immune cell surveillance against the peripheral tissues due to the BBB, which prevents circulating leukocytes from entering the CNS.^8^ Excessive immune responses can corrupt tight junctions (TJs) and BECs in brain injury. Within this section, we describe the physiological and pathological changes of different BBB components after ICH (summarized in Figure 1), in addition to immune responses and their associated effects on these cells.

### 2.1 Brain endothelial cells

#### 2.1.1 Pathological changes in BECs after ICH

BECs line cerebral vessels and form a continuous monolayer, which in contrast to the periphery, has no fenestrations and is particularly tight due to the expression of TJPs and adhesion junctions.^9^ Research reported that BECs can undergo cell death after ICH in experimental rodents and in vitro models.^10^ BBB impairments have also been observed in larger animal models, such as sheep.^11^ Erythrocyte lysis within ICH leads to hemoglobin release, degradation, and subsequent iron release, contributing to BBB breakdown. Oxidized heme and iron are pivotal in damaging BECs by promoting lipid peroxidation, which can alter BBB function.^12^

In our previous studies in experimental ICH in mice, BECs became swollen and irregular in shape, the TJs were shorter and blurred, and the expression of TJPs was significantly lower, with their lowest expression on day 3 after ICH (investigated time window for up to 5 days).^13^, ^14^ Using gadolinium‐enhanced T1‐MRI and Evans Blue (EB) staining, we observed that the breakdown of the BBB began to recover on day 7.^15^ Interestingly, a decrease in BBB extravasation has been observed in different models of ICH, including the autologous blood model^14^ and the thrombin model,^16^ indicating that the BBB may have the potential for repair after injury. Currently, changes at the BBB during the recovery period of ICH remain poorly understood, and research on the changes in BECs and TJPs during the repair stage is warranted.

The paracellular pathway, a crucial mechanism involving alterations in the function of TJs, is extensively studied for its role in regulating barrier permeability after ICH. Understanding the impact of changes in the number of blood vessels after ICH on TJPs expression is vital. This requires evaluating whether TJP reduction is linked to a decrease in the number of blood vessels, a comparison that can be facilitated by considering TJPs with vessel length.

In addition to the well‐documented role of the TJs network in governing the BBB through the paracellular route,^17^ BEC endocytosis emerges as a critical, yet less explored, mechanism in modulating BBB function after ICH. Studies have indicated a significant reduction in the expression of Mfsd2a, a transmembrane protein facilitating the cellular transport of BECs, in the brain tissue surrounding the hematoma after ICH. The findings illustrate that the overexpression of Mfsd2a can suppress vesicle‐mediated endocytosis, lower the number of vesicles, and attenuate BBB impairment after ICH.^18^ However, ongoing research aims to delineate the alterations in transporters at the BBB, including SLCs, SLC7A5, LAT1, or ABC efflux pumps, after ICH.

#### 2.1.2 The immunological impact of ICH on the BECs

BECs play a crucial role in maintaining CNS homeostasis by limiting the entry of circulating WBCs and blood molecules into the CNS.^19^ Following ICH, peripheral immune cells infiltrate the surrounding tissues of the hematoma through the damaged BBB. The expression of inhibitory MHC class I, specifically the ligand H2‐Kb, in BECs is downregulated, while ligands that activate natural killer cells (NK cells) such as RAE1 and MULT‐1 increase, which may lead to cell apoptosis in BECs.^20^ There are also reports that antigen presentation and recognition in MHC class I induces CD8 activity, leading to BBB breakdown.^21^ Suggests that the interaction between BECs and peripheral immune cells may exacerbate the disruption of the BBB after ICH. In addition, NK cells also significantly increase the mRNA expression of chemokines and cytokines that act on BECs, such as chemokine (C‐X‐C motif) ligand 2 (CXCL2), CXCL9, chemokine (C‐C motif) ligand 2 (CCL2), tumor necrosis factor (TNF), and interleukin‐6 (IL‐6).^20^ Therefore, blocking the inflammatory response can effectively prevent the degradation of TJs and protect the integrity of the BBB.^22^, ^23^, ^24^ In summary, the excessive inflammatory response of BECs after ICH can cause BECs damage and exacerbate the disruption of the BBB.

### 2.2 Astrocytes

Astrocyte end feet covers much of the cerebrovascular network and is in close contact with the BBB. They can release neurotrophic factors and metabolites, maintain the dynamic balance of molecules and water, and contribute to the formation and regulation of the BBB.^24^ After cerebral ischemia, due to the inability of blood to flow back to astrocytes, the end feet of astrocytes swell and reduce contact with the BM, leading to damage to the BBB.^25^ Although it was found that astrocytes have close contact with blood vessels through transmembrane anchoring proteins after ICH,^26^ the microscopic changes of astrocytes at the site of BBB injury have not been elucidated yet. The main water channel expressed in astrocyte end feet is aquaporin‐4 (AQP4). As the expression of AQP4 decreases in astrocytes after ICH,^27^ this may suggest that the tight contact between astrocyte terminals and blood vessels will also be disrupted after ICH.

Research suggests that inflammatory reactive astrocytes (A1 type) are essential in disrupting the BBB.^28^ Following ICH, astrocytes are also activated to reactive astrocytes, and the number of reactive astrocytes significantly increases on the first day after ICH and remains increased for 7 days.^29^ Astrocytes contribute to the damage of the BBB by releasing many cytokines. The expression of CCL5 is increased in astrocytes after ICH. Knocking out CCL5 on astrocytes reduces the infiltration of CD8^+^ cytotoxic T cells and increases the expression of TJPs, maintaining the integrity of BBB.^30^ In addition, astrocytes also express other inflammatory signals such as chemokine (C‐C motif) receptor 1, CCR1,^22^ CCR5,^31^ and nuclear factor‐κB (NF‐κB)^32^ after ICH. On the other hand, A2‐type astrocytes provide protective effects after ICH; they not only downregulate pro‐inflammatory factors but also improve neurological function in mice.^33^ However, whether they can affect BBB function remains unknown.

The functions of astrocytes are diverse, and recently it has been reported that astrocytes can also serve as compensatory phagocytic mechanisms for microglia^34^ This means that dividing astrocytes into A1 and A2 phenotypes is limited. In short, there is some evidence that the proinflammatory response of astrocytes further worsens BBB damage following ICH. Still, the different subtypes of astrocytes after ICH and their impact on the BBB have not yet been determined.

### 2.3 Pericytes

Pericytes are contractile cells that wrap around the BECs of capillaries, and they are particularly abundant in the brain's microvasculature. Pericytes interact with BECs, astrocytes, microglia, and other cells to maintain BBB integrity.^25^ Following ICH, the separation of pericytes from the endothelial membrane leads to a significant loss of pericytes, evidenced by the reduced expression of its markers pericytes‐derived platelet‐derived growth factor receptor beta and NG2.^35^ Pericytes accumulate a large amount of Fe^2+^ and even die due to the influence of excessive oxidative stress after ICH, leading to BBB damage, suggesting that inhibiting oxidative stress response can improve pericyte survival.^36^ Additionally, pericytes are also very sensitive to thrombin following ICH. Pericytes release MMP‐9^37^ upon thrombin stimulation, which may exacerbate BBB impairment after ICH, as discussed above, but some studies also suggest that thrombin stimulation can increase the coverage of pericytes.^38^ Therefore, like astrocytes, pericytes may be affected by immune inflammatory reactions, stimulating inflammatory pathways and influencing BBB function after ICH. The precise mechanisms and the role of pericytes in BBB breakdown should be further investigated.

### 2.4 Basement membrane

The BM is a thin, specialized extracellular matrix that serves as a structural foundation and support for BECs and epithelial cells. It is separated by pericytes into parenchymal BM and endothelial BM.^39^ Mural cell‐specific knockout of the BM protein laminin led to an increase in BBB permeability after experimental ICH in mice,^40^, ^41^ suggesting an essential role of the BM for BBB function. In line with this, lysine hydroxylase 3 (LH3), an enzyme that catalyzes the formation of the BM protein collagen, is reduced in the plasma of ICH patients.^42^ In experimental ICH in mice, LH3 mRNA expression significantly decreased starting on the third day after ICH, and LH3 overexpression enhanced the integrity of the BM. This also correlated with a dampened inflammatory response after LH3 overexpression.^42^

## 3 THE ROLE OF MICROGLIA/MACROPHAGES AND PERIPHERAL IMMUNE CELLS ON THE BBB IN ICH

**FIGURE 2:**
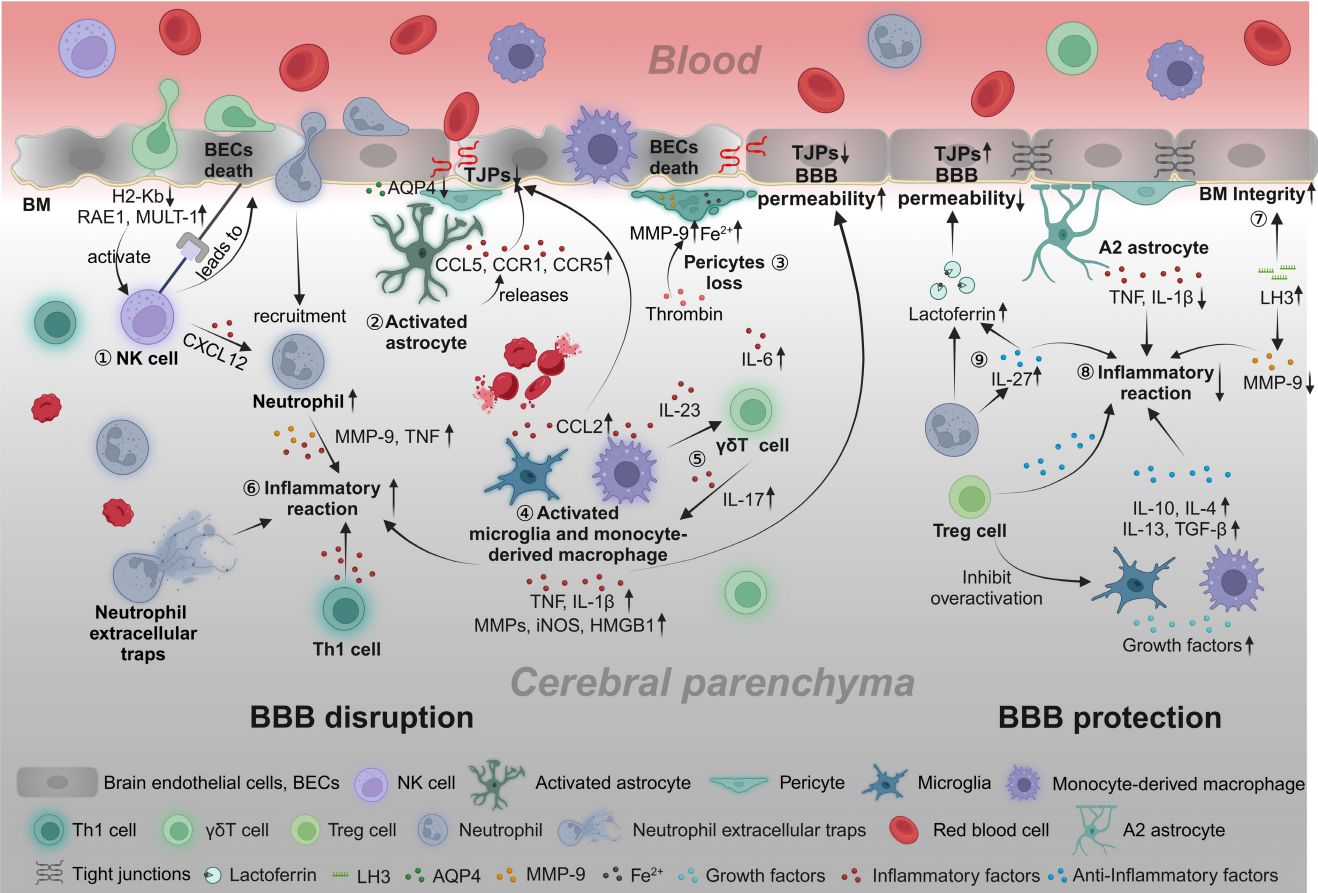
Interactions between immune cells and the BBB after ICH. Detrimental mechanisms: ① Downregulated H2‐kb and upregulated RAE1 and MULT‐1 in BECs activate NK cells, which mediate BECs death. NK cells produce CXCL12, enhancing neutrophil recruitment. ② Astrocytes are activated to reactive astrocytes, releasing CCL5, CCR1, and CCR5, downregulating TJPs to disrupt the integrity of the BBB. ③ Fe^2+^ accumulates in pericytes, which are stimulated by thrombin and release MMP‐9, exacerbating BBB damage. ④ Activated M/M express CCL2 and reduce the expression of TJPs. ⑤ IL‐23, secreted by macrophages, stimulates γδT cells to produce IL‐17, which activates macrophages. ⑥ Neutrophils, neutrophil extracellular traps, Th1 cells and, activated M/M increase pro‐inflammatory factors such as IL‐1β, TNF, and inflammatory damage molecules such as MMPs, iNOS, HMGB1, which exacerbate inflammation and increase BBB permeability. Protective mechanisms: ⑦ LH3 overexpression enhances the integrity of the BM and decreases MMP‐9 expression. ⑧ Immune cells also release a large amount of anti‐inflammatory factors to help repair the BBB. A2‐type astrocytes reduce the expression of IL‐1β and TNF. Treg cells play an immunosuppressive role by inhibiting M/M overactivation. ⑨ IL‐27 production by neutrophils is increased, which has led to an increase in the production of the iron scavengers lactoferrin, promoting the clearance of Fe^2+^ and the integrity of the BBB (Created with BioRender.com).

After ICH occurs, the initial responders are microglia, followed by infiltrating macrophages appearing within minutes to hours around the hematoma. Subsequently, neutrophils peak at day 3 post‐infarction and persist until day 7, after which their numbers decline. Non‐conventional innate‐like T cells, such as γδT cells and CD8^+^ cytotoxic T cells, also migrate to the lesion site early on, with the arrival time of CD4^+^ T cells resembling neutrophils. These immune cells are involved in the complex pathological process of ICH, and excessive activation of immune cells and the release of inflammatory cytokines can lead to deterioration of the BBB.^43^, ^44^ Therefore, a thorough understanding of the cellular and molecular immune components that affect the BBB in the hemorrhagic brain may help identify potential therapeutic targets for ICH. The current knowledge of the causes and mechanisms by which immune cells lead to BBB disruption is summarized in Figure 2.

### 3.1 Microglia/macrophages (M/M)

Microglia, the primary immune cells in the CNS, quickly react to hemorrhagic damage and are the initial responders to extravasated blood within 1–1.5 h post‐ICH.^45^ Due to the disruption of the BBB, peripheral macrophages also infiltrate around the hematoma, and they jointly regulate BBB function after ICH. The efficacious elimination of the hematoma is critical for mitigating damage to the BBB and facilitating recovery from ICH. This importance stems from the fact that hematomas contain substances potentially deleterious to neuronal cells, such as iron and by‐products of hemoglobin degradation. The removal of these toxic substances aids in reducing further harm to the BBB. M/M can engulf and digest hemoglobin and other blood components in hematoma, playing a crucial role in clearing the hematoma.^46^, ^47^

Subsequently, the accumulation of activated M/M at the injury site can directly affect the permeability of the BBB. M/M causes local inflammation and neuronal degradation by releasing pro‐inflammatory factors, for instance, IL‐1β, TNF, and express chemokines such as CCL2 and chemokine (C‐X3‐C motif) receptor 1 (Cx3cr1) that activate BECs, alter BBB permeability, and increase the recruitment of white blood cells (WBCs). M/M also expresses inflammatory damage molecules, including MMPs, inducible nitric oxide synthase (iNOS), and high mobility group box 1 (HMGB1), exacerbating BBB damage after ICH.^48^ Moreover, BBB damage is repaired 2 weeks after bleeding,^46^ which is related to mechanisms such as anti‐inflammatory effects, tissue repair, and angiogenesis. The anti‐inflammatory factors released by M/M increase during the disease recovery stage, including IL‐10, IL‐4, and transforming growth factor beta (TGF‐β), which have been associated with improvements in the function of the BBB by inhibiting inflammatory reactions and enhancing hematoma clearance.^49^, ^50^ Meanwhile, M/M also expresses growth factors related to tissue and inflammation recovery, such as vascular endothelial growth factor, platelet‐derived growth factor, and brain‐derived neurotrophic factor, promoting BBB repair.^51^

In summary, M/M plays an important role in regulating the BBB after ICH. However, further exploration is needed to unravel the similarities and differences in the effects of M/M on the BBB.

### 3.2 Lymphocytes

Lymphocytes, including T cells, NK cells, and B cells, are essential immune system components that play complex roles in ICH.

Emerging evidence indicates a dynamic interplay between BBB dysfunction and lymphocyte infiltration in ICH. T cells contribute intricately to the inflammatory environment, with substantial evidence indicating their pro‐inflammatory impact within the brain after ICH.^52^, ^53^ Furthermore, studies involving T‐cell‐deficient nude mice and those treated with siponimod^54^ and fingolimod^52^ exhibit decreased BBB leakage post‐ICH. Gene expression analysis revealed that γδ T cells and NK cells were enriched, whereas natural regulatory T cells were lower after ICH than the control group.^44^ In ICH mouse models, CD3^+^ T cell levels peak on day 5, aligning with maximum BBB permeability evidenced by EB leakage and a significant drop in TJPs.^52^ Th1 cells exacerbate BBB damage by secreting inflammatory cytokines like IL‐12.^55^ NK cells can aggravate brain injury within 12 hours following ICH. When NK cells are inhibited, there is a reduction in EB penetration and BEC damage, while the expression of TJPs such as claudin‐5 and ZO‐1 is enhanced.^20^ NK cells induce cytotoxic effects on BECs while recruiting neutrophils via CXCL12, exacerbating BBB damage and increasing brain edema.^56^ Treg cells exhibit anti‐inflammatory effects on microglia following ICH,^57^ though their influence on the BBB remains unexplored. Therefore, suppressing detrimental lymphocyte responses may serve as a strategy for preserving BBB function post‐ICH. In forthcoming research, it will be essential to delve into the specific cell types and mechanisms through which lymphocytes impact the BBB following ICH.

### 3.3 Neutrophils

Neutrophils are a type of WBC renowned for their swift reaction to infections and inflammation. Neutrophils in the CNS challenge the traditional notion that the BBB acts as an impenetrable barrier against immune cells. An upregulation of genes associated with neutrophils was observed in ICH patients.^58^, ^59^ After ICH in rodent models, an increase in neutrophil levels is often accompanied by further disruption of the BBB.^20^ Depleting neutrophils in experimental ICH leads to a reduction in BBB permeability, MMP‐9 levels, axonal injury, as well as the activation of astrocytes and M/M following ICH.^57^, ^60^ During the initial week following ICH, activated neutrophils that cling to BECs generate neutrophil extracellular traps, composed of active proteases and condensed chromatin, intensifying inflammation and encouraging thrombosis.^61^ Furthermore, NETs hinder the resolution of hematomas and exacerbate BBB leakage by impairing the fibrinolytic activity of tissue plasminogen activator.^61^ Moreover, neutrophils have the potential to foster the formation of blood clots within cerebral vessels, thereby exacerbating the situation.^62^, ^63^

On the other hand, it has also been reported that neutrophils can play protective roles. For example, the release of lactoferrin, which scavenges iron, contributes to decreasing perihematomal edema (PHE) and enhancing the clearance of the hematoma. IL‐27 decreases the presence of inflammatory/cytotoxic molecules in the neutrophil secretome and increases the expression of lactoferrin, alleviating oxidative stress after ICH.^64^ Notably, this process is systemic, as the alteration in the neutrophil secretome occurs in the bone marrow.

Comprehending the intricate interplay between neutrophils and the BBB following ICH is imperative for developing precise therapeutic interventions.

## 4 THE INFLUENCE OF CYTOKINES ON BBB

Many recent studies have demonstrated that neuroinflammation is a risk factor and leads to BBB dysfunction in various brain diseases.^65^ The characteristic of inflammation is the accumulation and activation of inflammatory cells and mediators in the hemorrhagic brain, which begins cytokine production mostly by activating leukocytes, astrocytes, and microglial cells in the brain.^16^ A class of polypeptides known as cytokines is implicated in inflammatory reactions, so the prognosis of patients can be judged by the characteristic changes of these cytokines. To better understand the mechanisms involving immune responses at the BBB following ICH, we review recent research on the impact of cytokines on BBB function.

### 4.1 Pro‐inflammatory cytokines

#### 4.1.1 IL‐1β

IL‐1β is an inflammatory cytokine predominantly generated by activated glial cells and monocytes.^66^ Extensive research has revealed a marked surge in IL‐1β release following ICH as an integral component of the inflammatory cascade.^16^ IL‐1β instigates many inflammatory pathways, notably the activation of NF‐κB facilitated by its receptor interactions.^55^ This activation, in turn, incites the migration of immune cells and sets off a robust inflammatory reaction. The impact of IL‐1β on the integrity of the BBB represents a pivotal facet of the inflammatory response following ICH. It was closely linked to the occurrence and exacerbation of PHE, contributing to the mortality and disability observed in animal models. Decreasing IL‐1β levels pharmacologically has been associated with less BBB impairment and improved expression of occludin and claudin in ICH mice.^67^, ^68^, ^69^ We encountered IL‐1β as a biomarker for detection rather than a direct influencing factor, and the precise pathway through which IL‐1β exerts its function remains elusive. It is plausible that its primary role may be within the resident brain cells rather than the infiltrating peripheral cells.^70^

#### 4.1.2 TNF

TNF is released by various cells, including microglia and astrocytes, and triggers an inflammatory cascade that plays a pivotal role in the inflammatory response following ICH.^65^ TNF protein levels increase rapidly, often within just 6 h, in the areas surrounding the hematoma. These levels continue to rise over time, persisting for at least 72 h after ICH.^55^ TNF can traverse the BBB through receptor‐mediated endocytosis and interact with BECs, decreasing BBB stability.^71^ After ischemic stroke, microglia are the primary source of TNF secretion. This secretion can result in BEC death by necroptosis, but treatment with the anti‐TNF antibody infliximab has demonstrated significant improvements.^72^ However, whereas BECs are known to be injured after ICH, there has been no direct investigation of the impact of TNF on BBB injury after ICH. Recent studies indicate a correlation between increased TNF levels and more severe cerebral edema following ICH.^73^, ^74^ This may suggest more extensive BBB damage, although the exact mechanisms remain unclear. This underscores the idea that different cytokines may target distinct cells or tissues, prompting further exploration into the specific functions of TNF secreted by various cell types.

#### 4.1.3 IL‐6

IL‐6 is a promising biomarker for predicting the risk and evaluating the severity of ICH. IL‐6 is a proinflammatory factor, and its concentration in the tissue surrounding the hematoma typically rises during the acute stages following an ICH in mice.^75^ Moreover, its concentrations in cerebrospinal fluid and blood correlated with the degree of PHE and the prognosis of individuals affected by hemorrhagic strokes.^76^, ^77^ Some studies have shown that an improvement in BBB impairment after ICH is accompanied by a decrease in the expression of IL‐6,^78^, ^79^ and the direct effect of IL‐6 on BBB injury has been suggested in other models.^80^ However, it is still unknown in the ICH. It has also been reported that IL‐6 can induce an M2‐type macrophage phenotype, contributing to an anti‐inflammatory response.^81^ Hence, we cannot just consider the pro‐inflammatory effects of IL‐6; we need to explore its potential role in the BBB after ICH from multiple perspectives.

#### 4.1.4 IL‐17/IL‐23

The IL‐23/IL‐17 axis is crucial in the inflammatory response after ICH. Specifically, IL‐23 and IL‐17 are upregulated in the vicinity of the hematoma. IL‐23 reaches its peak at day 1, while IL‐17 peaks at day 4.^82^ Notably, IL‐17 has been reported to be neurotoxic post‐ICH and to enhance autophagy and inflammatory responses of microglia through IL‐17 receptors.^83^ Furthermore, the level of IL‐17 in the serum of ICH patients is negatively correlated with neurological function recovery.^84^

Another aspect to consider is the potential influence of cytokines on cell interactions after ICH. It has been demonstrated that IL‐23, secreted by macrophages, can stimulate T cells to produce IL‐17, thereby exacerbating PHE post‐ICH.^82^ This finding suggests that cytokines significantly mediate cell‐to‐cell communication, which can greatly impact brain injury. Consequently, future research should delve further into elucidating the specific effects of cell interactions through cytokines on the integrity of the BBB following ICH.

### 4.2 Anti‐inflammatory cytokines

Only a few studies have examined the impact of anti‐inflammatory cytokines on the BBB after ICH. TGF‐β1 administration can reduce PHE, improve BBB integrity, activate anti‐inflammatory microglia, and enhance long‐term outcomes in an ICH model, suggesting TGF‐β1 as a potential therapeutic agent for BBB after ICH.^85^ IL‐10 was found to be correlated with relative PHE volume in the cerebrospinal fluid of patients with ICH.^76^ IL‐10 can regulate the inflammatory response of M/M after ICH in mice^86^ and alleviate inflammation and cell death by inhibiting the level of nerve growth factor precursor, thereby protecting brain tissue after ICH.^87^ Other published reports indicate that IL‐10 increases TJPs and improves BBB permeability in acute pancreatitis,^88^ but its specific mechanism in ICH is still unclear. Understanding the intricate interplay between anti‐inflammatory cytokines and the BBB after ICH is essential for developing therapeutic strategies to mitigate the deleterious effects of neuroinflammation and promote recovery.

### 4.3 Chemokines

Chemokines are small cytokines or signaling proteins secreted by cells. They can induce targeted chemotaxis of cells and play a pivotal role in inflammatory responses. After ICH, immune cells release numerous chemokines, remarkably increasing the expression levels of CCL and CXCL families.^89^ The greater the concentration of these chemokines, the more pronounced their impact on BBB permeability.^31^

CCL2 has been identified as a factor contributing to BBB damage after ICH. Increased CCL2 protein expression has been associated with reductions in TJP expression, an exacerbation of PHE, and an increase in BBB permeability.^90^, ^91^ CCR1 also plays a leading role in promoting inflammation.^92^ Inhibiting CCL5 can counteract the impact of CCR1, and blocking CCR1 improves the integrity of the BBB.^22^ CCL17‐dependent CCR4 activation can mitigate PHE after ICH.^93^ This effect may be attributed to the more substantial chemotactic impact of CCR4 on Treg cells compared to other cell types, thereby exerting an immunosuppressive role. CCR5 has been associated with neuronal damage after ICH, although its documented impact on the BBB remains unestablished.^94^

CXCL2 is upregulated in NK cells after ICH, which enhances the recruitment of neutrophils and exacerbates damage to the BBB.^20^ Additionally, CXCL12 enhances the proliferation, migration, adhesion, and vascular repair capabilities of BECs after ICH.^89^ CCL20 emerges as an inflammatory biomarker following ICH, providing a promising target for anti‐inflammatory interventions.^95^ The levels of several other members of the CXCL family, including CXCL9, CXCL11, CXCL17, and CXCL21, also increased after ICH,^20^ though research on these factors remains limited.

In summary, chemokines can respond to inflammation and alter the immune milieu surrounding the hematoma by influencing cell migration, particularly impacting WBCs. Nonetheless, further studies should be performed beyond expression detection to investigate the specific mechanisms of their effects as a basis for developing targeted treatments.

### 4.4 Adhesion molecules

Adhesion molecules produced by BECs play a crucial role in facilitating the migration of leukocytes across the endothelium and are closely associated with the maintenance of BBB function.^96^ When WBCs recognize the blood vessel wall through signal molecules like selectins, the interaction between WBC adhesion molecules and vascular endothelial cell adhesion molecules results in firm adhesion of WBCs to BECs. Subsequently, they can infiltrate the CNS, recognize antigens in the brain, and release numerous cytokines and chemokines, inciting an inflammatory response.^8^ Decreasing levels of intercellular adhesion molecules^90^ and vascular endothelial cell adhesion molecules^97^ can enhance BBB integrity and reduce PHE. α4 integrin was elevated in all leukocyte populations in the brain after ICH, which reduces leukocyte migration into the brain, diminishing neurobehavioral disability.^98^ However, the role of immunoglobulin family members and selectins in BBB function after ICH remains undefined, with the specific types of WBCs requiring further clarification. Additionally, pro‐inflammatory factors have been shown to influence BBB permeability and the expression of adhesion molecules.^96^

### 4.5 Matrix metalloproteinases

MMPs are a family of proteases that participate in physiological and pathophysiological processes. Elevated MMP levels disrupt the normal metabolism balance of the extracellular matrix, resulting in the degradation of blood capillary BM and TJ structures. This degradation, in turn, leads to increased BBB permeability and clinical hematoma expansion.^99^ Consequently, inhibiting MMP activation mitigates BBB damage following ICH.^100^, ^101^

MMPs, particularly MMP‐2 and MMP‐9, have been implicated in the breakdown of BBB integrity, contributing to increased permeability and PHE. This process is closely tied to the recruitment and activation of immune cells, including neutrophils and macrophages, which release pro‐inflammatory factors and chemokines. These immune responses and MMP activity further exacerbate BBB disruption and tissue damage. Previous findings have indicated increased MMP‐9 levels in peripheral blood are associated with hematoma enlargement, poor functional prognosis, and increased PHE growth in patients.^102^ Inhibiting MMP‐9 can increase the expression of TJPs and basal protein.^40^ However, some studies have suggested that MMP‐9 activation may not be a contributing factor to the neurological outcomes in hypertension‐induced ICH.^103^ Additionally, distinct members within the MMPs family may exhibit different effects. For example, MMP‐2 shows no independent correlation with early hematoma expansion in ICH patients^104^ but is associated with ICH recurrence.^105^ Notably, in this study, no indicators were linked to the BBB. In future research, a more comprehensive exploration of MMP‐2 and the impact of other MMPs family members on the BBB, possibly in conjunction with clinical detection methods, is warranted.

## 5 THERAPEUTIC ADVANCES TARGETING IMMUNOREGULATORY EFFECTS TO ALLEVIATE BBB IMPAIRMENT AFTER ICH

The primary approach to managing hemorrhagic stroke is typically a craniotomy intended to stop the bleeding or reduce intracranial pressure. However, these interventions are associated with several challenges, including constraints related to the time window for intervention, suboptimal efficacy, a heightened risk of complications, and limitations in terms of patient eligibility. Currently, novel treatment strategies targeting BBB dysfunction have garnered increasing recognition in the ICH research field. Numerous drugs and gene targets have demonstrated efficacy in promoting BBB recovery, presenting promising avenues for therapeutic interventions. The potential targets for BBB protection post‐ICH are summarized in Table 1.

**TABLE 1:** Potential targets for BBB protection post ICH.

Pre‐clinical studies						
Targets	Known to	Method or effect	Cell type	Function	Mechanism	Reference
H2‐kb	MHC‐I ligand, a ligand for the inhibitory NK cell receptor Ly49C	Protective effects	BECs	Prevent apoptosis of BECs	–	[20]
Laminin α5	Laminin	Protective effects	BECs	① Reduce BBB permeability ② Reduce neuronal death ③ Reduce inflammatory response	–	[134]
MMP‐9	Matrix metalloproteinases	Inhibit	BECs and BM	Improve expression of TJ proteins and basal proteins	–	[40]
AQP4	Family of membrane channel proteins	Protective effects	Astrocytes	Improve BBB integrity and Peri‐hematoma edema	Relates to micro‐environmental reactive oxygen species	[27]
CCL5	Chemokine ligand	Inhibit	Astrocytes	Reduce the infiltration of CD8^+^ cytotoxic T cells Reduce the loss of TJPs, maintaining the integrity of BBB	–	[30]
LH3	An enzyme that catalyzes the formation of the BM protein collagen	Protective effects	BM	① Enhance the integrity of the BM ② Attenuate neuroinflammation	–	[42]
TSG‐6	Tumor necrosis factor 6	Protective effects	Astrocytes	Antagonize the inflammatory response of activated astrocytes	Inhibits NF‐κB pathway	[32]
NDP‐MSH	Nle4‐D‐Phe7‐α‐MSH, transforming from α‐MSH	Protective effects	Microglia	① Lessen BBB leakage ② Increase the expression of ZO‐1, occludin, and laminin‐α5 ③ Attenuate neuroinflammation	Activates CREB/Nr4a1/NF‐κB pathway	[23]
Didymin	A dietary citrus flavonoid	Protective effects	Microglia	① Attenuate neuroinflammation ② Decrease BBB disruption and brain water content	Upregulates Rkip expression and suppresses the Asc/Caspase‐1/GSDMD signal pathway	[69]
P7C3‐A20	A novel aminopropyl carbazole compound	Protective effects	Microglia	① Lessen BBB leakage ② Reverse the reduced protein levels of TJs ③ Attenuate neuroinflammation	Activate the NAD^+^/Sirt3 Pathway	[110]
Soluble epoxide hydrolase	The key enzyme in the Epoxyeicosatrienoic acid signaling	Inhibit	Microglial Neutrophils	Induce neuroinflammatory responses	–	[111]
CCR1/CCL2	C‐C chemokine receptor/Chemokine CC ligand 2	Inhibit	BECs Astrocyte NeuN	① Reduce the BBB permeability ② Reverse the reduced protein levels of TJs	① Inhibit CCR1/SRC/Rac1 signal pathway ② Inhibit CCL2/CCR2/p38 MAPK signal pathway	[22, 91]
Heat shock protein B8, HSPB8	Small heat shock proteins family	Protective effects	BECs	① Reduce the brain water content ② Lessen BBB leakage ③ Reduce damage of TJs	Activate the Akt/GSK3β/β‐catenin pathway	[135]

Clinical studies						
Targets	Known to	Phase	Cell type	Function	Mechanism	
Dexmedetomidine	A2‐adrenergic receptor agonist	Phase II	Microglia	Reduce the volume of hematoma, PHE, and inflammatory reaction	① Inhibit the activation of NLRP3 ② Suppress NF‐κB signal pathway	[107, 136, 137]
Methylprednisolone sodium succinate	An immunosuppressive agent	Pre‐clinical	Microglia Neutrophils	① Reduce permeability of the BBB and PHE ② Inhibit inflammatory responses ③ Reduce neuronal apoptosis	① Attenuate activation of the TLR4/NF‐κB pathway ② Increase Bcl‐2 expression and reduce Bax expression	[108]
Minocycline	An antibiotic	Phase II and III	Microglia	Alleviate BBB damage, PHE, neurological deficit, and inflammatory reaction	Alter the activity of DKK1‐Wnt Signaling	[101, 106, 138]
Ozanimod	Sphingosine‐1‐phosphate (S1P) 1/5 receptor agonist	Pre‐clinical	Microglia Lymphocyte	① Improve the neural function ② Reduce the number of activated microglia and neutrophils	S1PR regulator	[109]
Fingolimod	A sphingosine 1‐phosphate analog	Phase II/0	Lymphocyte	Regulate the integrity and reduce permeability of the BBB	S1PR regulator	[139]
Glibenclamide	Sulfonylurea	Phase II	BECs Microglia	Reduce the brain water content, BBB permeability, and inflammatory reaction	Activate NLRP3 inflammatory bodies	[140]
Deferoxamine	Iron chelator	Phase II	BECs	Reduce the inflammatory activation	–	[141, 142]

Relieving excessive immune cell proliferation and activation is a way to deal with the inflammatory reaction brought on by BBB breakdown after ICH. Compounds like Nle4‐D‐Phe7‐α‐MSH,^23^ minocycline, dexmedetomidine, methylprednisolone, Didymin,^69^ ozanimod, and P7C3‐A20 can inhibit microglial activation and alleviate inflammatory reactions through various pathways.^106^, ^107^, ^108^, ^109^, ^110^ Some have also demonstrated effects on astrocytes, neutrophils,^69^ and peripheral infiltrating macrophages.^107^ Research on targeting lymphocytes in the context of BBB disruption after ICH is relatively limited. While siponimod^54^ and fingolimod^52^ have shown potential in reducing lymphocyte infiltration, its exact molecular mechanism of action remains unknown.

In addition, reducing pro‐inflammatory factors and chemokines is also an important strategy to reduce immune cell infiltration and help with regression. Inhibitors targeting inflammatory factors and chemokines, such as CCR1 and CCL2 receptor inhibitors,^22^, ^91^, ^111^ primarily enhance BBB function after ICH by reducing the expression of proinflammatory cytokines and chemokines. However, the effectiveness of these inhibitors often depends on the activation state of cells.

Cell transplantation technology holds significant potential for improving BBB function. Pericytes from pluripotent stem cells reduce post‐stroke BBB permeability in vivo by increasing coverage on BECs and the expression of TJPs.^112^ In the context of ICH, studies involving cell transplantation, such as mesenchymal stromal cell transplantation, have also been reported to improve neurological function after ICH.^113^, ^114^ However, we emphasize the need for more integration of tissue engineering methods in these approaches. Over recent years, emerging tissue engineering has provided potential therapeutic strategies for BBB recovery after ICH, including the application of biomaterials in drug delivery and cell transplantation. Hydrogel, a biological material derived from various polymers, exhibits excellent permeability and accuracy across the BBB. Hydrogel with drug therapy has demonstrated a therapeutic effect in improving inflammatory reactions and reducing PHE after ICH.^115^, ^116^, ^117^ Hydrogel delivers Procyanidins, which can more effectively reduce reactive oxygen species levels.^118^ Nanomaterials utilizing carbon clusters containing high iron chelators can effectively reduce oxidative stress.^119^ The 3D scaffold constructed with graphene enhances adhesion proteins' adhesion and facilitates nerve cell regeneration and development.^120^ However, all of the above biomaterials have their limitations, such as dosage volume, time, efficacy, and injection method, which need to be considered.^121^ In this regard, we hypothesize that optimizing nasal delivery and intravenous injection methods may greatly improve clinical translation. 3D tissue engineering technology aims to create three‐dimensional structures that mimic the morphology and function of biological tissues in vivo.^122^ Both hydrogel and graphene scaffolds can be used as a platform for attaching immune cells and other elements involved in the recovery of neural function. In vitro experiments have successfully utilized these biomaterials in combination with 3D printing technology to generate cerebral micro‐vessels or even complete neurovascular units, simulating the BBB.^123^, ^124^, ^125^ The combination of biomaterials and immune cells has been studied in other fields,^126^ but research on ICH has not been reported yet. A study showed that microglia have characteristic morphology, inflammatory reaction, phagocytosis, and other functions in 3D organoids,^127^ suggesting that 3D tissue engineering technology and cell transplantation technology can be combined to study the function of the BBB after ICH.

## 6 CONCLUSIONS AND PERSPECTIVES

The immune response following ICH is a complex process triggered by multifaceted factors. The BBB, a crucial boundary between the central and peripheral areas, becomes a target for these immune responses. While studies have provided a comprehensive overview of the inflammatory response post‐ICH,^16^ the specific impact of the immune response from distinct BBB components on ICH remains unclear. This underscores the necessity for in‐depth research on the changes and mechanisms of the immune responses to BBB components.^128^ This understanding is paramount as the morphology and structure of BECs undergo significant changes after ICH,^14^ leading to functional damage.

The increased expression of MHC ligand^20^ on BECs enhances the antigen presentation of BECs. It facilitates the recognition by immune cells and their transport to the CNS after ICH. Similarly, the increased expression of chemokines and adhesion molecules^20^ is crucial in recruiting immune cells to BECs. After ICH, astrocytes transform to an inflammatory phenotype,^29^ releasing proinflammatory factors that induce BEC activation. Meanwhile, the inflammatory response is more likely to result in passive loss of pericytes and BM after ICH. Therefore, any disruption of these components inevitably leads to BBB dysfunction.

Brain‐resident microglia are the primary responders to ICH, followed by peripheral macrophages, lymphocytes, and neutrophils that gradually infiltrate the lesion over several days.^129^, ^130^ Microglia, in particular, can lead to BBB destruction by releasing numerous inflammatory factors in the early stage of ICH.^48^ Eliminating microglia has been shown to improve the prognosis of ICH.^131^ Therefore, understanding the protective effects of microglia and their proinflammatory role in the early stage of BBB destruction is of utmost importance. Macrophages, on the other hand, gradually transform toward the phenotype of microglia after entering the brain. It is crucial to distinguish the effects of microglia and peripherally infiltrating macrophages on the BBB after ICH, yet many studies do not differentiate them. There is no study on brain‐resident macrophages on the BBB, highlighting a significant research gap. In addition, the metabolic environment varies in different disease environments, and M/M are important sources of metabolites.^132^ Whether the metabolites of M/M have an impact on the BBB function after ICH is also a new research direction.

The time point of infiltration of lymphocytes and neutrophils after ICH is inconsistent, possibly due to the differential expression of BEC adhesion molecules, selectin ligands, etc., causing variations in different infiltrating cell types.^133^ Peripheral infiltrating lymphocytes and neutrophils are believed to have a destructive effect on the BBB.^53^ However, the exact function and mechanism of action of different types of immune cells in BBB damage after ICH are still a puzzle. There are numerous unresolved issues, such as the differences in the effects of various subtypes of lymphocytes on the BBB after ICH, the interaction between peripheral infiltrating immune cells and brain cells to affect BBB function after ICH, and their role in the recovery phase of the BBB after ICH. These gaps in knowledge underscore the need for multi‐angle interpretation and in‐depth exploration of the mechanism of the effects of different immune cells on the BBB after ICH in future research.

The interaction between central and peripheral immune cells emerges as a vital factor in damaging and protecting the BBB after ICH. This review synthesizes the current understanding of how ICH influences immune responses within various BBB components and underscores the significant yet often underestimated effects of peripheral immune cells infiltrating the BBB. Furthermore, it discusses current therapeutic approaches targeting the inflammatory responses at the BBB post‐ICH, thus paving the way for future investigative avenues.

## AUTHOR CONTRIBUTIONS

Peijun Jia, Qinfeng Peng devised the conceptual ideas, performed the literature search, and drafted the original manuscript. Yumeng Zhang, Hanxiao Xue, Jiaxin Li, and Houn Sonita assisted with writing and formatting modifications. Xiaochong Fan, Junmin Wang, Simon Liu, Anh Le, Shijie Zhang, Marietta Zille, Chao Jiang, Xuemei Chen, and Jian Wang contributed to the discussion and revision. All authors approved the final manuscript.

## FUNDING INFORMATION

This work received partial support from the National Natural Science Foundation of China (82371339).

## CONFLICT OF INTEREST STATEMENT

The author declares no conflict of interest.

## Data Availability

Data sharing is not applicable to this article, as no datasets were generated or analyzed during the current study.
